# Costs of health care across primary care models in Ontario

**DOI:** 10.1186/s12913-017-2455-1

**Published:** 2017-08-01

**Authors:** Maude Laberge, Walter P Wodchis, Jan Barnsley, Audrey Laporte

**Affiliations:** 1 Department of Operations and Decision Systems, Faculty of Administrative Sciences, Université Laval, 2325 rue de la Terrasse, #2519, Quebec City, G1V0A6 Quebec Canada; 2 Canadian Centre for Health Economics, Toronto, Canada; 3 Institute of Health Policy, Management and Evaluation, University of Toronto, Toronto, Canada; 4 Institute for Clinical Evaluative Sciences, Toronto, Canada; 5 Toronto Rehabilitation Institute, Toronto, Canada

**Keywords:** Health care costs, Primary care, Payment systems: FFS/capitation/DRGs/Risk Adjusted Payments etc.

## Abstract

**Background:**

The purpose of this study is to analyze the relationship between newly introduced primary care models in Ontario, Canada, and patients’ primary care and total health care costs. A specific focus is on the payment mechanisms for primary care physicians, i.e. fee-for-service (FFS), enhanced-FFS, and blended capitation, and whether providers practiced as part of a multidisciplinary team.

**Methods:**

Utilization data for a one year period was measured using administrative databases for a 10% sample selected at random from the Ontario adult population. Primary care and total health care costs were calculated at the individual level and included costs from physician services, hospital visits and admissions, long term care, drugs, home care, lab tests, and visits to non-medical health care providers. Generalized linear model regressions were conducted to assess the differences in costs between primary care models.

**Results:**

Patients not enrolled with a primary care physicians were younger, more likely to be males and of lower socio-economic status. Patients in blended capitation models were healthier and wealthier than FFS and enhanced-FFS patients. Primary care and total health care costs were significantly different across Ontario primary care models. Using the traditional FFS as the reference, we found that patients in the enhanced-FFS models had the lowest total health care costs, and also the lowest primary care costs. Patients in the blended capitation models had higher primary care costs but lower total health care costs. Patients that were in multidisciplinary teams (FHT), where physicians are also paid on a blended capitation basis, had higher total health care costs than non-FHT patients but still lower than the FFS reference group. Primary care and total health care costs increased with patients’ age, morbidity, and lower income quintile across all primary care payment types.

**Conclusions:**

The new primary care models were associated with lower total health care costs for patients compared to the traditional FFS model, despite higher primary care costs in some models.

## Background

Physician payments and methods of remuneration have been topics of increasing interest as policy makers search for the “right” payment policy to balance physicians’, patients’, and payers’ interests [1]. Physicians may be incentivized to provide fewer or more services depending on the payment methods, yet how sensitive they are to the financial incentives may depend on their level of altruism [2].

In most countries, payers also have a responsibility towards maintaining and improving the health of the population within budget constraints. In this context, payers – in many cases, governments – introduced different ways of remunerating physicians, particularly in primary care. One example is the Quality and Outcomes Framework (QOF) in the UK, a program that blends capitation payment with incentives and rewards for primary care physicians to meet performance targets. Most of these targets are related to the management of common chronic conditions, and the delivery of preventive services [3]. The implementation of a new remuneration scheme for primary care physicians such as the QOF is typically associated with an increase in the spending in primary care.

The evidence suggests that the processes of strengthening primary care with measures including higher investments and chronic disease management programs would be more than compensated for by avoidance of a population’s deterioration in health and of complications that result in higher utilization of expensive health care services [4–8].

In Canada, where the responsibility for health care is decentralized to the provinces, the government of Ontario (the most populous province) initiated a reform of primary care in the early 2000s. The reform consisted of the gradual introduction of models characterized by mixed payment mechanisms as well as practice requirements and an enrollment process, formally defining the physician-patient relationship [9]. The reform had multiple aims including improving access to and quality of primary care and controlling health care costs. Primary care services include diagnostic and treatment for the majority of health conditions, patient education, health promotion and disease prevention services. These services are mainly delivered by family physicians, nurses, and pharmacists. In Canada, family physicians are the entry point into the health care system and act as gatekeepers to access to specialized services; primary care services are meant to provide the majority of health care needs to the population. The context at the time was one of primary care physician shortage, where Ontarians reported experiencing difficulties finding a primary care physician, and when they had one, being able to see their physician in a timely matter [10, 11]. The vast majority of primary care physicians were remunerated on a fee-for-service (FFS) basis. The FFS method is considered to have important drawbacks not only because it provides an incentive to over-supply care, but also, because remuneration (at least in Ontario) is disconnected from the quality of care provided and may inhibit collaboration amongst health care providers [12, 13]. On the other hand, FFS physicians provide more services than physicians paid by capitation or salary [14], which should be associated with better access.

Between 2001 and 2007, the Ontario government introduced new primary care models offering physicians the option of shifting away from FFS towards enhanced-FFS and blended capitation payment mechanisms. Both of these payment mechanisms consisted of mixing FFS payment and capitation payment with bonus and incentive payments for the delivery of preventive services (such as cancer screening) and chronic disease management (such as for diabetes).

There are currently two enhanced-FFS models: the Comprehensive Care Model (CCM) and the Family Health Group (FHG); the latter requires physicians to practice in a group of at least three physicians. Because of the differences in payment and practice structures, CCM and FHG are considered separately. There are also two blended capitation payment models: the Family Health Network (FHN) and the Family Health Organization (FHO). FHOs have a higher capitation rate than FHNs, which reflects a larger basket of primary care services included in the rate. Services provided outside of the basket are compensated via FFS; FHN and FHO physicians also receive 15% of the fees for services that are included in the capitation rates. In addition, FHOs and FHNs can apply to the Ontario Ministry of Health and Long Term Care (MOHLTC) to form a Family Health Team (FHT). FHTs receive additional funding to hire other types of health care professionals and support an interdisciplinary team environment. There are no intended differences in physicians’ payment whether the practice is a FHT or not. Because the payments are different between FHO and FHN, they are considered separate primary care models. Each of the FHO and FHN models are also distinguished by whether they practice as a FHT.

In the Canadian context where health care services are provided free at the point of services, it is assumed that the utilization of health care services is determined by one’s needs, as opposed to ability to pay. In general, older individuals and individuals in poorer health have a higher utilization of health care services. Others have shown that a lower socio-economic status (SES) is associated with a higher utilization, even after adjusting for health status [15].

This study examines the associations between the primary care model a patient belongs to, and patients’ primary care costs and total health care costs, using the traditional FFS model as the reference.

## Methods

All Ontario residents (13.6 million people in 2013) are covered by the Ontario Health Insurance Plan (OHIP) for medically necessary services. Our cohort population for this study consisted of a 10% random sample of the adult population who had a valid health care card at the index date (April 1, 2012). Individuals were excluded from the study sample if they incurred zero primary care costs (meaning that they did not use physician services) or if they died during the study period (April 1 2012 to March 21st 2013).

### Data source and variables

Encrypted data for this cross-sectional study were obtained from administrative databases at the Institute for Clinical Evaluative Sciences (ICES). The study received ethics approval from the Research Ethics Boards of the University of Toronto and the Sunnybrook Health Sciences Centre. The sample was extracted from the adult population in the Ontario Registered Persons Database (RPDB) which contains basic demographic information on individuals. Using unique patient identifiers, the ICES Key Numbers (IKN), patients’ enrolment data at the beginning of the study period from the Client Agency Program Enrolment (CAPE) database were used to link patients to their primary care physicians and to the corresponding payment model to which the physician belonged. Patients who were not formally enrolled with a physician were considered as a separate group – the not enrolled. The IKN were also used to retrieve each individual’s health services utilization. All health care services paid for by the MOHLTC were included: physician services were extracted from the OHIP database; general hospital services from the Canadian Institute for Health Information Discharge Abstract Database, the Same Day Surgery Database, and the National Ambulatory Care Reporting System; drugs for adults on social assistance and for people aged 65 and over from the Ontario Drug Benefit Plan database; home care services from the Home Care database; lab and non-physician billings from the OHIP. Utilization from long term episodes of care were combined to include residents of nursing homes and of specialized hospitals with data from the Complex Continuing Care database, the Ontario Mental Health Reporting System database and the Long Term Care database.

The two outcome variables were primary care costs (PCC) and total health care costs (THCC).

Costs were calculated for each individual in the study population based on the individual’s utilization of health care services during the study period and the prices of services, as paid by the MOHLTC. Algorithms for the prices of health care services have been previously developed by a team of Ontario researchers and implemented at ICES [16]. Primary care costs were composed of the prices and quantity of each service billed by primary care physicians to OHIP as well as capitation costs, and shadow billing costs. The capitation rates depended on the primary care model that the physician belonged to.

Total health care costs were calculated based on the utilization of health care services that each individual made. They included services paid for on a fee schedule as well as institutional care. Costs for institutional care were adjusted for the resource intensity weight of the care setting. In the case of long term care, costs were based on a per diem fee paid by the MOHLTC.

The costs associated with the payments for the establishment and operations of FHTs, as well as performance payments to primary care physicians were not available for inclusion at the individual patient level.

The independent variables of interest were the primary care models that physicians can belong to: CCM, FHG, FHN, FHO, FHT-FHN, FHT-FHO, using the FFS as the reference group.

Since patients may be differentially distributed across models [17], the statistical models controlled for patient characteristics that could affect health care utilization and costs. Explanatory variables included patient’s age (continuous variable), sex (dichotomous variable), and socio-economic status, using the income quintile as a proxy (based on postal codes with the lowest quintile (1) as the reference group), the Adjusted Clinical Group (ACG®) weight, and the Rurality Index of Ontario (RIO) score for the practice location of the primary care physician.

The ACG® system is a measure of case-mix developed by the Johns Hopkins University to reflect patients’ health care needs based on the combination of their diagnostics, age and sex [18]. The ACG® weight ranges from 0.000 to 4.666, with a higher weight representing a poorer health status. There are only a few scores over 1.000, and these reflect a high level of complexity with a combination of: at least 2 major adjusted diagnostic groups (ADGs); at least 6 other ADG combinations; and an age over 34. The algorithm accounts for the mix of diagnosis over a defined period of time and across health care settings. The ACG® has been tested and it was validated as a predictor of utilization and mortality in Canada [19–21]. A recent review of various morbidity measures suggests that the ACG® system is the strongest in predicting health care utilization [22]. The RIO is a continuous variable that takes a value between 0 and 100, with lower values indicating an urban location and it adjusts for the geographic location of the primary care practice. The RIO is a measure that was developed by the Ontario Medical Association for Ontario communities [23]. The RIO includes the following 10 variables: travel time to nearest basic referral center, travel time to nearest advanced referral center, community population, number of active general practitioners (GP), population-to-GP ratio, presence of a hospital, availability of ambulance services, social indicators, weather conditions, and selected services.

### Statistical Analysis

There are multiple approaches to the analysis of cost data, with the most common being the OLS with a log transformation and the generalized linear model [GLM] [24–28]. Although the OLS with log transformation is widely known and used, it does not eliminate heteroscedasticity and the retransformation could lead to bias. The GLM is a preferred approach in the presence of heteroscedasticity in a statistical model with multiple covariates. The GLM was selected for this study after the Breusch-Pagan test found heteroscedasticity and because of other advantages of the GLM. The GLM takes into account heteroscedasticity, and does not require retransformation so as to express estimates in dollar amounts and accommodates skewness, which is typical of health care cost data [29–31]. This method does require specifying a distribution for the mean-variance relationship and a link function. Gaussian, Poisson, Gamma and inverse Gaussian distributions for the mean-variance relationship were tested with the modified Park test [24, 30]. The results reported here are based on the GLM with an identity link and a Gaussian family based on the results from the Park test.

Two regressions were conducted: the first regression modelled patient primary care costs, and dummy variables for the models; the second analysis examined total health care costs as a function of the primary care model of the primary care physician and also of the other explanatory variables identified.

Regression models for both primary care and total health care costs were defined as:

$$ {\mathrm{Cost}}_{\mathrm{i}}={\upbeta}_0+{\upbeta}_1\ {CCM}_{\mathrm{i}}+{\upbeta}_2\ {FHG}_{\mathrm{i}}+{\upbeta}_3\ {FHN}_{\mathrm{i}}+{\upbeta}_4{FHO}_{\mathrm{i}}+{\upbeta}_5\ FHT-{FHN}_{\mathrm{i}}+{\upbeta}_6\ FHT-{FHO}_{\mathrm{i}}+{\upbeta}_7{RIO}_{\mathrm{i}}+{\upbeta}_8\ {\mathrm{ACGweight}}_{\mathrm{i}}+{\upbeta}_9\ {\mathrm{age}}_{\mathrm{i}}+{\upbeta}_{10}\ {\mathrm{sex}}_{\mathrm{i}}+{\upbeta}_7\ \mathrm{income}\_{\mathrm{quintile}}_{\mathrm{i}}+{\upvarepsilon}_{\mathrm{i}} $$

Where: Cost_i_ is either the primary care or the total health care cost of the services for patient i for a 12 month period; β_0_ is the intercept; CCM_i_, FHG_i_, FHN_i_, FHO_i_, FHT-FHN_i_,and FHT-FHO_i_ are dichotomous variables for the primary care models, using FFS as the reference group; RIO_i_ is the value of the RIO of the practice the patient i belongs to; age_i_, sex_i,_ and income_quintile_i_ are the adjustments for the patient’s age, sex, and neighborhood income quintile; ɛ_i_ is the error term for patient i.

## Results

The sample contained 1,133,645 observations. Table 1 describes the characteristics of the patients in the sample as a whole and according to primary care model, including the average costs, broken down by health care sector and types of services.

**Table 1:** Comparison of Patient Characteristics and Costs between FFS and each other Primary Care Model

Variable	Total	FFS (reference)	Not enrolled	CCM	FHG	FHN	FHO	FHT-FHN	FHT-FHO
N	1,133,645	90,112	96,945	37,554	345,783	5691	338,345	25,767	193,448
Mean patient age (sd)	39.8 (22.3)	33.7 (23.7)	35.9*** (21.9)	41.7*** (21.6)	39.9*** (21.6)	42.4*** (23.2)	41.5*** (22.3)	40.4*** (23.0)	41.0*** (22.6)
% Female	52.7	50.9	50.6	52.4***	53.5***	52.9**	52.7***	53.0***	53.3***
Mean ACG® weight (sd)	0.521 (0.711)	0.575 (0.835)	0.513*** (0.716)	0.550*** (0.709)	0.546*** (0.703)	0.523*** (0.767)	0.504*** (0.691)	0.480*** (0.690)	0.486*** (0.690)
Mean RIO (sd)	8.0 (14.6)	5.0 (12.8)	6.5*** (13.5)	8.7*** (16.3)	3.5*** (8.0)	34.0*** (16.9)	8.6*** (14.3)	37.6*** (25.9)	11.9*** (16.2)
% Income quint 1	18.1	22.3	21.2***	20.9***	18.3***	15.4***	16.1***	18.9***	17.0***
% Income quint 2	19.3	20.7	19.6***	22.4***	19.8***	17.6***	18.3***	20.6	18.9***
% Income quint 3	20.2	19.2	20.0***	20.7***	21.1***	19.9	19.5	18.8	20.2***
% Income quint 4	21.7	19.8	20.3**	20.0	22.0***	23.3***	22.2*	20.3	22.0***
% Income quint 5	20.4	17.5	18.5***	15.6***	18.5***	23.6***	23.6***	20.5***	21.5***
Mean Hospital Costs: SDS, inpatient, cancer, ED, dialysis (sd)	$765 (4895)	$873 (5765)	$825 (5312)	$791* (5660)	$676*** (4544)	$989 (4595)	$770*** (4772)	$906 (4796)	$802** (4908)
Mean Physician costs (sd): OHIP billings, shadow billings, capitation	$690 (1228)	$716 (1374)	$672*** (1286)	$676*** (1197)	$687*** (1222)	$664*** (1092)	$706* (1239)	$608*** (1045)	$679*** (1143)
Mean Long Term Episodes Costs (sd): CCC, LTC, OMHRS	$163 (2552)	$177 (2670)	$303*** (3524)	$271*** (4540)	$228*** (3739)	$174 (2599)	$154*** (2464)	$207 (2901)	$159* (2523)
ODB Cost (sd)	$318 (2065)	$319 (1737)	$324 (4180)	$332 (1400)	$300** (1730)	$389** (1804)	$320 (1570)	$361** (1840)	$333 (2057)
NRS Cost (sd)	$34 (1059)	$33 (1006)	$32 (1040)	$27 (820)	$30 (924)	$43 (1378)	$38 (1196)	$29 (1462)	$35 (1030)
HC cost (sd)	$119 (1389)	$147 (1890)	$127* (1446)	$105*** (1211)	$103*** (1369)	$141 (1236)	$119*** (1253)	$137 (1338)	$132* (1390)
Lab costs (sd)	$66 (109)	$66 (112)	$67 (112)	$74*** (112)	$75*** (116)	$43*** (87)	$63*** (105)	$48*** (97)	$58*** (102)
Non MD costs (sd)	$22 (133)	$22 (140)	$25*** (161)	$21 (122)	$20*** (124)	$27* (151)	$23 (134)	$24* (128)	$23 (132)
Mean Total health system costs (sd)	$2300 (8848)	$2531 (10,015)	$2608 (11,175)	$2297*** (9441)	$2119*** (8100)	$2612 (8665)	$2304*** (8524)	$2427 (8684)	$2334*** (8685)
Mean Primary Care costs (sd)	$288 (416)	$274 (605)	$268* (532)	$269 (372)	$278 (426)	$305*** (378)	$307*** (349)	$297*** (377)	$293*** (333)

*ACG®* Adjusted Clinical Group, *CCC* Complex Continuing Care, *CCM* Comprehensive Care model, *ED* Emergency Department, *FFS* Fee-For-Service, *FHG* Family Health Group, *FHT* Family Health Team, *FHN* Family Health Network, *FHO* Family Health Organization, *HC* Home Care, *LTC* Long Term Care, *NRS* National Rehabilitation Reporting System, *ODB* Ontario Drub Database, *OHIP* Ontario Health Insurance Plan, *OMHRS* Ontario Mental Health Reporting System, *RIO* Rurality Index of Ontario, *SDS* same-day surgery

Significantly different from FFS at: *p < 0.05, **p < 0.01, ***p < 0.001

The highest health care costs are hospital costs, followed by physician costs. The results in Table 1 show that there are significant differences in the characteristics of patients across models compared to FFS patients and significant differences in the costs as well.

The sample size was reduced to 1,094,687 because of missing data on the RIO variable for patients across all models. The marginal effects in dollars of the explanatory variables were computed from the GLM models, using the method described by Jones et al. (2013) and are reported in Table 2. The marginal effects assessed at the mean of the explanatory variables are reported below.

**Table 2:** The Average Marginal Effect of Primary Care Models on One-Year Primary Care Costs and on Total Health Care Costs

Variable	Primary Care Cost (in $)	Total Health Care Cost (in $)
*N* = 1,094,687		
Practice Characteristics:		
FFS	reference	reference
Not enrolled	−5	130*
Enhanced FFS – CCM	−32***	−658***
Enhanced group FFS - FHG	−13**	−667***
FHN	0.1	−446***
FHO	16***	−485**
FHT – FHN	2	−433***
FHT - FHO	5	−392***
Practice RIO	0.3***	6.8***
Patient Characteristics:		
Patient age	4***	61***
Patient female	48***	−101***
ACG® weight	128***	2947***
Income quintile 1	Reference	Reference
Income quintile 2	−17***	−325***
Income quintile 3	−21***	−436***
Income quintile 4	−27***	−517***
Income quintile 5	−33***	−607***

***indicates significance at *p* < 0.001; **indicates significance at *p* < 0.01; *indicates significance at *p* < 0.05

The effect of the primary care model on a patient’s costs is measured through the variables CCM, FHG, FHN, FHO, FHT-FHN, and FHT-FHO. Compared to FFS patients, primary care costs for one year are on average $32 lower for CCM patients and $13 lower for FHG patients. They are $16 higher for FHO patients. The primary care costs of patients who are not enrolled but who are seeing a physician who is in a patient enrolment model are not significantly different from those of patients seeing FFS physicians. Compared to FFS patients, total health care costs are lower on average by $658, $667, $446, $485, $433, and $392 for CCM, FHG, FHN, FHO, FHT-FHN, and FHT-FHO patients respectively. They are $130 higher for not-enrolled patients.

Although the ACG® weight, age and sex control for a patient’s health status, the SES of a patient still has a significant effect on both primary care costs and total health care costs, which are incrementally lower with higher SES, as measured with the neighborhood income quintile.

## Discussion

The results suggest there are statistically significant differences in both total health care and primary care costs across primary care models after controlling for patient health status and SES as well as practice location. The discussion is structured as follows: first, a discussion of the primary care costs; then, a discussion of the total health care costs; finally a review of some limitations of the study that may explain some of the results obtained.

### Primary care costs

The lower primary care costs of enhanced-FFS patients ($32 lower for CCM patients and $13 lower for FHG patients) appear counter-intuitive to the fact that the payment mechanisms are relatively similar, in that CCM and FHG are based on FFS payment. It may be that the small incentives for enrolment of patients in CCM and FHG, as well as the incentives for preventive services and chronic disease management are effective at supporting higher quality of care, continuity of the patient-physician relationship and that these indirectly support lower primary care needs and visits compared to the FFS patients [32]. Non-enrolled patients had primary care costs that were not significantly different from those of FFS patients. This was an expected result in that payments to physicians for these patients are the same as for patients of FFS physicians. Because these patients were not enrolled to the physician that they were seeing, they were maybe also less likely to have a continuous relationship with the physician and a monitoring and management of their health.

FHO patients had primary care costs that were $16 higher than FFS patients. These higher primary care costs for patients in a blended-capitation models could appear counterintuitive in relation to the theoretical literature, which supports capitation remuneration as a form of cost containment [33]. But this significant difference in primary care cost can be explained by a policy objective associated with the FHO model of moving physicians away from the FFS payment model. To attract physicians into a prospective and risk-sharing payment mechanism, the remuneration had to be higher than the expected remuneration on a FFS basis, all else being equal. The higher adjusted per-patient costs reflect that the FHO payments are in fact higher than what would have been paid to primary care physicians in the FFS model. The FHN was a blended capitation model introduced before the FHO and it was seen as unsuccessful; few physicians formed FHNs because the capitation rates were considered too low [34]. The results in Table 2 in regards to FHN patients are consistent with the idea that the FHN capitation rates were not high enough to entice physicians to join or form FHNs: primary care costs of FHN patients are not significantly different from those of FFS patients.

Finally, the primary care costs of FHT patients, both FHT-FHNs and FHT-FHOs were not significantly different from those of FFS patients. These results may reflect the fact that the additional funding provided by the MOHLTC for the establishment and operations of FHTs were not available for inclusion. The funding supports the hiring of various health care providers, which may substitute for some of the care that would be delivered by physicians in non-FHT practices. Hence the primary costs of FHT patients were under-estimated in our analysis and the observed higher costs, could be significant if the additional funding were included. In 2014, the average operational budget of FHTs was $1.75 million with a range from $0.27 million to $21.7 million, and these budgets were considered as having been stable in the past few years. The total budget from the MOHLTC to FHTs was $344 million [personal communication, Association of Family Health Teams of Ontario, January 18th, 2016 & Ministry of Health and Long Term Care, Primary Health Care Branch, January 21st, 2016]. The MOHLTC also reports that over 3 million patients are enrolled in FHTs. Based on these numbers from the total budget and the number of patients enrolled, a rough estimate of the additional FHT per patient cost would be $114.67. Hence, it could be extrapolated that the primary care costs of FHT patients are likely to be significantly higher than those of FFS patients.

Primary care costs increased with higher rurality of the primary care practice, with a patient’s age, and higher morbidity reflecting higher needs in older and sicker patients. Being female was associated with on average $48 higher primary care costs also reflecting the higher medical needs of women. Results also showed an income gradient in that primary care decrease with higher income neighborhood quintile. Higher utilization of primary care services in lower income population, even after adjusting for morbidity has been observed elsewhere in Canada [15]; it may partly be attributable to the lower health literacy and capacity to benefit from a physician visit in patients of lower SES [35].

### Total health care costs

Total health care costs were significantly lower in patients of all primary care models compared to FFS patients. The not enrolled patients had higher total health care costs.

Compared to FFS, the fact that total health care costs were lower in blended capitation model patients may appear surprising: the theoretical literature suggests that a capitation payment to primary care physicians provides an incentive for an under-provision of care and a shift towards other levels of care that are more expensive, such as specialists’ care or hospital care [33]. In fact, Allard et al. (2011) suggest that FFS would be the optimal payment system if primary care physicians all had a high diagnostic ability and were relatively altruistic in their desire to provide high-quality care, or if patients’ outcomes were measured and considered in the payments. However, it is more likely that there is heterogeneity amongst physicians in relation to those two aspects. In addition, in a context such as the one in Ontario, where primary care physicians have various payment options, physicians may choose the primary care model that is most beneficial in relation to their skills and altruism levels, given the characteristics of their patient population (which they can also choose). In fact, other researchers who examined physicians’ levels of altruism found heterogeneity and differences in optimal payment methods, depending on a physician’s altruism [36–39]. It has also been found that quantities of care provided were closer to the optimum for subjects with higher levels of altruism and that mixed payment alleviated the negative effects associated with the incentives in each type of payment mechanism [2].

Although the purpose of this study was to analyze the costs using the traditional FFS as the reference group, examining the results between the models reveals some interesting findings. Within models, the differences in total health care costs were higher in enhanced-FFS models, i.e. the CCM ($-658) and the FHG ($-667) patients whereas the total health care costs of patients in blended capitation models were lower than those of FFS patients but not by as much: $-446 for FHN patients, $-485 for FHO patients, $-433 for FHN-FHT patients, and $-392 for FHO-FHT patients. These results are consistent with those of a study where patients of enhanced-FFS physicians had the lowest potentially preventable hospital utilization, followed by patients of physicians in blended capitation models [40]. The fact that total health care costs are the lowest in the enhanced-FFS models is consistent with the idea that physicians’ levels of altruism and ability may not be randomly distributed across primary care models.

Including the estimated $114.67 additional cost per FHT patient to the patients’ total health care costs would reduce the differences between FFS patients and FHT patients (FHT-FHO and FHT-FHN). However, FHT patients are likely to still have significantly lower total health care costs than FFS patients. The FHTs were designed to offer more comprehensive services with a range of services to more complex patients. However, our results, adjusting for patient characteristics suggest that although FHTs appears to be cost saving compared to FFS patients, FHT patients are not less costly than patients in other primary care models. It may be that there is a misalignment between the characteristics of the patients treated in the FHTs and the services offered. The results in Table 1 do show that the average ACG® weight of FHT patients is significantly lower compared to the average of FFS patients and it is also lower than the averages of patients in all other models. The income quintile distribution shows that FHT patients are also wealthier.

In terms of control variables, the effects observed in primary care costs are accentuated in total health care costs except for the sex variable. As expected, total health care costs increased with practice rurality, with the patient’s age and ACG® weight, and decreased with higher SES. Surprisingly, being female was associated with lower total health care costs. It may be that women use more preventive primary care and, when controlling for morbidity with the ACG®, are better able to manage their health conditions than males.

The large effect of SES (on average, a $607 lower total health care cost for a patient from the highest neighborhood income quintile compared to the total health care cost of a patient from the lowest neighborhood income quintile) may reflect that people of lower SES have a lower health literacy and capacity to manage their health. It may also be that the ACG® does not accurately capture the severity of conditions and the severity of diseases afflicting people in lower SES is higher.

### Limitations

The limitations of this study include those related to outcomes examined and the availability of the data. Specifically, because we could only obtain limited data on the additional funding provided to FHTs, we could only provide estimates as an average for the patient level. Drug costs only included those paid for by the MOHLTC for: people 65 and older, residents of long-term care facilities or a home for special care, and people receiving social assistance, and people registered through the Trillium Drug Program (eligible only if their drug costs are considered high relative to their income). Primary care funding related to performance targets were not included in patient level costs and but these would affect costs across the primary care models to a similar extent, with the exception of the FFS patients, for which physicians do not receive any bonus payments.

Even though total health care costs at the patient level were found to be lower in the new primary care models compared to the traditional FFS model, these would need to be further examined in relation to the health outcomes of the patients.

## Conclusion

Blended capitation models were associated with higher primary care costs. However, our results suggest that these were more than compensated for with lower total health care costs. More research is needed to better understand the causes for higher total health care costs associated with patients for whom physicians are paid on a FFS basis and to understand potential implications in terms of quality of care and patient outcomes.

## Abbreviations

ACG®: Adjusted Clinical Group
CAPE Client: Agency Program Enrolment
CCM: Comprehensive Care model
FFS: Fee-For-Service
FHG: Family Health Group
FHN: Family Health Network
FHO: Family Health Organization
FHT: Family Health Team
GLM: Generalized Linear Model
ICES: Institute for Clinical Evaluative Sciences
IKN: ICES Key Number
MOHLTC: Ministry of Health and Long Term Care
OHIP: Ontario Health Insurance Plan
PCC: Primary Care Costs
QOF: Quality and Outcomes Framework
RIO: Rurality Index of Ontario
RPDB: Registered Persons Database
SES: Socio-Economic Status
THCC: Total Health Care Costs
